# Gallbladder Carcinoma: Radical Surgery?

**DOI:** 10.1155/1990/64068

**Published:** 1990

**Authors:** E. Moreno Gonzalez

**Affiliations:** Digestive Surgery Department Instituto Nacional de la Salud 12 de Octubre Madrid 28041 Spain


*HPB Surgery*, 1990, Vol. 2, pp. 295–302
Reprints available directly from the publisher
Photocopying permitted by license only

© 1990 Harwood Academic Publishers GmbH
Printed in the United Kingdom

### HPB INTERNATIONAL

**EDITORIAL & ABSTRACTING SERVICE**

JOHN TERBLANCHE, EDITOR
Department of Surgery,
Medical School · Observatory 7925
Cape Town · South Africa
Telephone: (021) 47-1250 Ext. 229
Telefax (021) 47-8955

### GALLBLADDER CARCINOMA: RADICAL SURGERY?

### ABSTRACT

*Nakamura, S., Sakaguchi, S., Suzuki, S. and Muro, H. (1989) Aggressive surgery for carcinoma of the gallbladder. Surgery, 106, 467–473*

**Forty patients with gallbladder cancer were admitted to our institution in a 9-year period. For two patients with Nevin's stage 1 carcinoma who had undergone cholecystectomy, resection of the lower portion of the fourth and fifth segments of the liver and extrahepatic bile duct with dissection of lymph nodes was carried out as a second-stage operation. Thirteen patients with stage V carcinoma underwent extensive aggressive operations. Operative procedures comprised various types of liver resection with cholecystectomy and extrahepatic bile duct resection and wide lymph node dissection in all cases, portal vein reconstruction in 3, pancreatoduodenectomy in 3, partial colectomy in 3, and right nephrectomy in 1. The operative and in-hospital mortality rates were 0%. Two patients with stage 1 carcinoma are both doing well. Two patients with stage V carcinoma who underwent an extended operation are working without recurrence 7 years 8 months and 8 years 5 months after surgery. From our experiences we believe that long-term survival may be achieved by aggressive surgery if it is suitably indicated.**

### PAPER DISCUSSION

KEY WORDS: Gallbladder, carcinoma, liver resection

The surgical treatment for gallbladder cancer has evolved in the last twenty years from the resection of the tumour and adjacent tissues to a more academic and oncologic resection, including the right hepatic lobe, segment IV and part of segment I (extended right hepatic lobectomy); the resection includes the totality of the extra-hepatic biliary tract and the perivascular fatty tissue and lymph nodes. The results of this type of surgery were not very successful in relation to survival (average survival 6.5–11 months after surgery) or postoperative complications. At present, there is almost universal agreement, at least in Western countries, that cancer of the gallbladder with nodal or visceral metastases (bile ducts, liver, pancreas, duodenum, portal vein) is a non curable disease, for which surgery has, at best, only a paleiative effect. As in other cases of cancer of the gastrointestinal tract, if the tumour involves only the mucosal layer without infiltration of the muscularis mucosae, the five year survival after total or partial resection of the organ is near 100%; in these cases there is no need to resect adjacent organs or to re-operate if diagnosed after cholecystectomy.

The paper published by Satoshi Nakamura *et al.*, demonstrates the different results obtained in Japan when compared with Western countries after very extended liver resections, with lymphadenectomy of the nodes located far away from the anatomical region of the primary tumour. In their paper, employing a diagnostic protocol more extensive than that commonly used in Western countries, an exploratory laparotomy was avoided in only 5 of 40 patients (12.5%). The authors do not explain how many of the 20 patients considered non-resectable were laparotomized, which would be an important point to determine the efficiency of their diagnostic procedures. Seventeen patients were icteric, and although serum bilirubin levels were 1.2–18 mg/dl, they were all treated with transhepatic percutaneous drainage, which is controversial; the authors also stated that the diagnostic procedures were started after improvement of the jaundice, which is not common practice in the West. Biliary lithiasis was present in only 65% of the cases which is the lowest figure in the medical literature (80–100%).

As is frequent in many series, in two cases the diagnosis was made after surgery, and although the tumours were limited to the mucosal layer both patients were reoperated upon with resection of segment V and the distal portion of segment IV; in one of the cases there was infiltration of the liver tissue, an uncommon finding. Probably, most surgeons would not have reoperated on these cases, but the patients would have been followed periodically.

At present, in this condition, it is exceptional to perform extended right hepatic lobectomy including segment IV. It is even more controversial to extend the resection to the duodenum and pancreas (5 patients of a total of 13; 38.4%), portal vein (3 patients of 13; 23%), partial colectomy (23%) or left kidney resection (1 of 13; 7.4%). Doubts must increase if we consider the poor prognosis for patients in stage V of the disease (Nevin) who underwent two of these extrahepatic resections (38.4%). Most probably, these patients would not have had a resection in most medical centers of the world.

At this time, the mortality of extended resections is very low (0–10%), but the lack of mortality in their series is striking considering the extent of the resections, although the number of complications is above the accepted average (38.4%). The survival of patients with liver resections (16% after 7 years and 8 months for patients in stage V) was compared with that of non-resected patients (10% after two years). These figures do not permit any conclusion because in this group the tumour was more aggressive or more advanced at the time of diagnosis; the figures only demonstrate that, without resection, some patients can live for prolonged periods of time (60% of them had multiple hepatic metastases). On the other hand, if we separate out the two patients who, for unknown reasons, survived more than 7 $\frac{1}{2}$ years, then the other 11 patients died between 4 and 30 months (average survival 12 months), which is not significantly different to non resected patients; this is more clearly seen if we consider that 7 of 11 (63.6%) patients died between 4 and 12 months after surgery (average, 7 months). Recurrences of disease were always located in the abdominal cavity, and in 50% of cases in the same anatomical region; this fact has led some authors to suggest intraoperative radiation therapy, combined with the extended resection. At present, the value of this form of treatment is unproven.

My opinion is that this is a laudable effort in the treatment of a disease which had very little chance of being cured in stages further than I or II. Also, the results for stage V are too optimistic, and contrary to most published results. Readers should evaluate these results very critically and compare them with their personal experience.

E. Moreno Gonzalez, MD
Head Digestive Surgery Department
Professor of Surgery
Instituto Nacional de la Salud
12 de Octubre
MADRID 28041
SPAIN

### LIVER RESECTION WITH INFLOW OCCLUSION

### ABSTRACT

*Delva, E., Camus, Y., Nordlinger, B., Honnoun, L., Parc, R., Deriaz, H., Lienhart, A. and Huguet, C. (1989) Vascular Occlusions for Liver Resections — Operative Management and Tolerance to Hepatic Ischemia: 142 Cases. Annals of Surgery,* **209**, 211–218.

**The intra- and early postoperative courses of 142 consecutive patients who underwent liver resections using vascular occlusions to reduce bleeding were reviewed. In 127 patients, the remnant liver parenchyma was normal, and 15 patients had liver cirrhosis. Eighty-five patients underwent major liver resections: right, extended right, or left lobectomies. Portal triad clamping (PTC) was used alone in 107 cases. Complete hepatic vascular exclusion (HVE) combining PTC and occlusion of the inferior vena cava below and above the liver was used for 35 major liver resections. These 35 patients had large or posterior liver tumors, and HVE was used to reduce**

